# Effect of senescence on some apoptosis and oxidative stress markers in infertile normozospermic and oligospermic men: A cross-sectional study

**Published:** 2018-07

**Authors:** Mathias Abiodun Emokpae, Happy Ngozi Chima

**Affiliations:** 1 *Department of Medical Laboratory Science, School of Basic Medical Sciences, College of Medical Sciences, University of Benin, Benin City, Nigeria.*; 2 *Department of Chemical Pathology, Ahmadu Bello University Teaching Hospital, Zaria, Nigeria. *

**Keywords:** Apoptosis, Caspase 3, Cytochrome C, Male infertility

## Abstract

**Background:**

Male senescence may affect testicular function, sperm indices and generation of high levels of oxidants and apoptosis.

**Objective:**

This study evaluates the effect of male age on the expression of some apoptosis and oxidative stress markers in seminal fluid of males investigated for infertility in a tertiary health institution in Nigeria.

**Materials and Methods:**

In this cross-sectional study, 122 men aged 20-60 yr who were investigated for infertility and were stratified according to age into four groups. Seminal plasma caspase 3, cytochrome C, and total antioxidant capacity (TAC) were assayed by ELISA technique, while manual semen analysis was performed according to WHO standard.

**Results:**

Seminal caspase 3, and cytochrome C activity increased while TAC and sperm indices decreased with increasing age. Cytochrome C (r=0.288; p=0.002) and caspase 3 (r=0.250; p=0.05) correlated significantly with age in normospermia while cytochrome C (r=0.314; p=0.02), caspase 3 (r=0.268; p=0.05), TAC (r=-0.342; p=0.01) and morphology percentage (r=-0.414; p=0.002) correlated with age in oligospermic infertile males.

**Conclusion:**

The measured apoptotic markers increased with increasing age while TAC and sperm indices decreased with increasing age of subjects evaluated. Although the levels of measured apoptosis and oxidative stress markers correlated with age in normozospermia, the effect on sperm indices was severe among oligospermia compare to normozospermia. Therefore, these markers may be assayed in aged men attending fertility clinics.

## Introduction

The negative effect of age on fecundity is well known in females but the impact of senescence on male reproduction is under reported. Male senescence may affect testicular function (1), sex hormones, sperm indices (2, 3), sperm DNA integrity, telomere length (4), structure of chromosomes and epigenetic factors (5). These alterations may negatively impact reproduction in aged men leading to higher frequency of congenital birth defects, abortion and fetal deaths (6) as well as increased apoptosis. Studies have reported a decline in testicular function indicated by decreased level of testosterone and elevated levels of gonadotropins (1). Others have observed age-related decline in sperm density, percent morphology and chromosomal abnormalities. Epidemiological studies have suggested that increasing age in males may correlate with abortion and birth defects in progeny. Also the time to achieve pregnancy for men aged 50 yr and above was reported to be longer than for younger men. Sperm indices were consistently reported to decline with age. Sperm DNA integrity was also observed to be negatively affected by age (1). The possible causes of sperm DNA damage can be divided into intrinsic (defective maturation process of spermatozoa, oxidative stress, abortive apoptosis) and extrinsic factors (life style behaviors such as smoking, obesity, excessive alcohol and caffeine consumption as well as inadequately treated genital infections, medication and substance abuse) (7, 8).

One of the best biomarkers of apoptosis in somatic cells is the presence of elevated caspase 3 activity (1). Caspase 3 induces activation of caspase-activated deoxy-ribonuclease (DNA fragmentation factor 40) that plays vital role in the degradation of DNA. Caspase 3 is responsible for the final disassembly of cells by generation of DNA strands-break (8). The initiator caspases are activated by an apoptotic stimulus that triggers the apoptotic pathway and leads to the activation of caspase 3, the signal that marks the irreversible point in the apoptotic cascade. Another apoptotic event that occurs in the mitochondria is the release of cytochrome C which is a caspase activator. Cytochrome C is a haem protein located between the outer and inner membranes of mitochondria. It acts as an activator to caspase 3 upon release into the cytoplasm (8). 

The World Health Organization (9) has provided standardized parameters of healthy/normal spermatozoa characteristics to be considered when evaluating male factor infertility. The semen analysis results obtained in the laboratory is compared with the standardized semen parameters to aid diagnosis. Damaged to the spermatozoa DNA is a factor that can undermine male fertility potential, which is not routinely determined in most fertility laboratories in Nigeria. To the best of our knowledge, the effect of age on apoptosis has not been reported in infertile male in northern Nigeria since most of the studies in literature were conducted in Caucasians (3-6).

The objective of this study was to evaluate the effect of male age on the expression of apoptosis and oxidative stress markers in seminal fluid of males investigated for infertility in a tertiary health institution in Zaria, northern Nigeria.

## Materials and methods

**Subjects**

This is a cross-sectional prospective study. The study participants were evaluated for infertility and consisted of 122 males with age range 20-60 yr. The participants gave informed consent for their specimens to be used for the study. Those included in the study were grouped according to sperm concentrations (normospermia (sperm concentration >15 ×10^6^/mL and motility >32%), oligospermia (sperm concentration <15 ×10^6^/mL) and age stratification: 20-30, 31-40, 41-50 and 51-60 yr. 

The oligospermic infertile men were those with sperm concentration <15 ×10^6^/mL, and oligo-asthenozoospermia (sperm concentration <15 ×10^6^/mL and progressive motility <32%). The participants had a normal testicular volume without varicocele. Those with evidence of chronic diseases (such as cardiovascular disease and diabetes), use of chronic medications and exposure to gonadotoxins (such as alcohol and substance abusers, cigarette smoking and chemotherapeutic agents) were excluded from the study. They were excluded because these aforementioned conditions may affect the integrity of sperm DNA. The female partners of the infertile men were previously evaluated and diagnosed as potentially fertile by gynaecologists. 

**Semen preparation**

Semen samples were collected by self or assisted masturbation into sterile containers after a minimum of 3 days of sexual abstinence. The samples which were collected into containers, labeled with time of collection and number code were sent to the laboratory within one hr of collection. Any loss of fraction was reported by the subjects. 

**Semen analysis**

The freshly collected semen samples were examined for liquefaction at room temperature. Volume was measured and pH checked, sperm count, motility and morphology were assayed microscopically using WHO protocol (9). Liquefied semen samples were centrifuged at 3000 rpm for 5 min. Seminal plasma was aspirated into plain container and kept frozen until assays for cytochrome C, caspases 3 and total antioxidant capacity were done. Seminal plasma cytochrome C, caspase 3 and total antioxidant capacity were assayed by sandwiched ELISA technique using reagents supplied by Elabscience and Wkea Medical supplies corporation, China respectively.

**Determination of seminal plasma caspase 3**

Seminal caspase 3 was done by sandwich ELISA technique (ELISA kit supplied by Wkea Medical Supplies Corporation China) and the concentration in the sample was read by extrapolation from the standard curve. 

**Estimation of cytochrome C**

Seminal plasma or standards were added to the micro plates wells pre-coated with monoclonal antibody specific to cytochrome C and they combine with the specific antibody. Then a biotinylated detection antibody specific for cytochrome C (Cyt-c) and Avidin-Horseadish Peroxidase (HRP) conjugate were added to each micro plate well and was incubated. Unbound antigens were washed off. Then substrate solution was added to each well. Those wells that contain Cty-C biotinylated detection antibody and Avidin- HPR conjugate will appear blue in color. The enzyme-substrate reaction was terminated by the addition of a sulphuric acid solution and the yellow color was measured spectrophotometrically at 450nm using a microplate well reader. The concentration of cytochrome C in the sample was extrapolated from the prepared standard curve (ELISA kit supplied by Elabscience, China). 

**Determination of total antioxidant capacity**

The seminal plasma or standards were added to the micro plates wells pre-coated with monoclonal antibody specific for total antioxidant capacity. The antigen binds to the pre-coated well. Unbound antigens were washed off after incubation at 37^o^C for 30 min. A standard solution of horseradish peroxidase (HRP) polyclonal antibody specific for total antioxidant capacity was added to each well. Antibody-antigen-enzymes antibody complex is formed. Unbound detection antibody was removed by washing after incubation at 37^o^C for 30 min. Then substrate A and B were added and allowed to react with the antibody-antigen enzyme antibody complex over a short period (15 min at 37^o^C) for color development. 

The reaction was stopped by addition of stop solution and the yellow color was measured spectrophotometrically at 450 nm using a microplate well reader. The concentration of total antioxidant capacity in the sample was determined by extrapolation from the standard curve (ELISA kit supplied by Wkea Medical Supplies Corporation, China).

**Ethical consideration**

The protocol for the study was reviewed and approved by the ethics committee of the hospital (code ABUTHZ/HREC/08/2015 dated 8th September, 2015) and the participants gave informed consent for the specimens to be used for the study.

**Statistical analysis**

Data were analyzed using the SPSS software (Statistical Package for the Social Sciences, version 20.0, Chicago IL, USA) and results were presented as mean±SEM. The measured variables were compared using analysis of variance (ANOVA). Measured variables were correlated with age of the study participants using Pearson correlation coefficient. p≤0.05 was considered statistically significant.

## Results

The data from this study are presented in tables I-III. Table I shows the results of the measured variables in men evaluated for infertility but had normal sperm concentrations (normospermia). The levels of cytochrome C were significantly higher in men aged 41-50 yr (p=0.05) and 51-60 yr (p=0.02) than those below 40 yr. Seminal plasma cytochrome C levels correlated positively (r=0.288; p=0.002) with age.

Caspase 3 activity correlated positively (r=0.250; p=0.05) with age of study participants. Conversely, the levels of TAC decreased with increasing age of study participants and levels in men aged 51-60 yr were significantly lower than the other age groups. The negative correlation between TAC and age was insignificant (r=-0.186; p=0.18).

Table II shows the levels of measured parameters in oligospermic males of different age groups. The levels of cytochrome C were significantly higher in men aged 31-40 yr (p=0.04), 41-50 yr (p=0.04) and 51-60 yr (p=0.01) compared with men aged 20-30 yr. The observed levels correlated positively (r=0.314; p=0.02) with age. The caspase 3 activity correlated positively (r=0.268; p=0.05) with age of participants. 

The levels of TAC decreased progressively with increasing age with levels significantly lower in men aged 31-40 yr (p=0.01) and above 40 yr (p<0.001) compared with those aged 20-30 yr. 

**Table I:** Comparison of measured parameters in normospermic but infertile males stratified by age

**Measured parameters**	**Group 1** **(20-30 yr)**	**Group 2** **(31-40 yr)**	**Group 3** **(41-50 yr)**	**Group 4** **(51-60 yr)**
No of subjects	10	37	11	08
Cytochrome C (pg/mL)	68.76 ± 16.1^a^ (32.6-104.8)	71.0 ± 9.9^a^ (7.8-244)	165.24 ± 13.3^a^, ^b^ (51-1182)	114.9 ± 9.1^c^ (13.3-537)
Caspase 3 (ng/mL)	2.56 ± 1.41^a^ (1.15-3.98)	3.11 ± 0.5^a^ (0.72-17.0)	5.15 ± 0.5^b^ (1.6-13.5)	5.29 ± 0.06^b^ (1.22-25)
TAC (U/mL)	3.56 ± 1.15^a^ 0.38-43.8)	2.64 ± 0.44^a^ (1.34-6.70)	2.33 ± 0.46^a^ (1.18-5.87)	0.73 ± 0.32^b^ 0.41-1.05)
Sperm concentration (10^6^/mL)	116.2 ± 8.3^a^ (32-200)	123.8 ± 3.8^a^ (16.5-290)	90.8 ± 2.3^d^ (15-300)	78.2 ± 1.9^d^ (23-162)
Sperm motility (%)	78.0 ± 4.9^a^ (40-90)	66.4 ± 3.6^a^ (20-90)	72.7 ± 4.9^a^ (50-90)	55.0 ± 5.0^b^ (50-60)
Sperm morphology (%)	68.5 ± 5.2^a^ (25-90)	65.3 ± 2.6^a^ (10-95)	68.6 ± 3.1^a^ (10-95)	55.0 ± 1.5^b^ (40-70)

The data are expressed as mean±SEM

Statistical significance is represented as

as compared between each age group using ANOVA.

TAC= Total antioxidant capacity.

a : p>0.05;

b : p=0.048;

c : p=0.02;

d : p<0.001;

e : p=0.01

**Table II:** Comparison of measured parameters according to male groups stratified by age in oligospermia

**Measured variables**	**Group 1** **(20-30 yr)**	**Group 2** **(31-40 yr)**	**Group 3** **(41-50 yr)**	**Group 4** **(51-60 yr)**
No of subjects	09	29	15	03
Cytochrome C (pg/mL)	45.5 ± 13.5^a^ (10-90)	173.7 ± 18.0^b^ (20-1193)	131.6 ± 14.4^b^ (32.2-395)	255.3 ± 24.0^d^ (26-356)
Caspase 3 (ng/mL)	2.43 ± 0.7^a^ (0.8-6.4)	3.70 ± 0.83^a^ (1.1-7.12)	3.36 ± 1.3^a^ (1.22-7.50)	5.75 ± 0.4^c^ (0.9-34)
TAC (U/mL)	3.40 ± 0.81^a^ (1.32-8.90)	2.83 ± 1.0^a^, ^d^ (1.2-5.02)	1.85 ± 0.1^e^ (0.8-5.66)	1.85 ± 0.2^e^ (1.4-2.25)
Sperm concentration (10^6^/mL)	5.41 ± 0.8^a^ (6-11)	5.37 ± 0.38^a^ (1.2-8.0)	4.56 ± 0.74^a^ (1.4-9.1)	4.73 ± 0.1^a^ (1.6-6)
Sperm motility (%)	51.1 ± 11.6^a^ (20-80)	34.0 ± 6.2^a^ (18-50)	36.3 ± 6.5^a^, ^e^ (15-60)	6.3 ± 0.1^e^ (1-7)
Sperm morphology (%)	52.3 ± 5.0^a^ (40-70)	42.2 ± 4.0^a^ (20-60)	46.7 ± 3.3^a^ (10-90)	40.0 ± 6.4^a^ 20-60)

The data are expressed as mean±SEM

Statistical significance is represented as

compared between each age group using ANOVA.

TAC= Total antioxidant capacity.

a : p>0.05;

b : p=0.04;

c : p=0.02;

d : p=0.01;

e : p<0.001,

**Table III:** Correlation of measured variables with age in normospermic and oligospermic subjects

**Parameters**	**R-value**	**p-value**
Normospermic subjects		
Cytochrome C and age	0.288	0.002
Caspase 3 and age	0.250	0.05
Total antioxidant capacity and age	-0.186	0.18
Sperm concentration and age	-0.177	0.10
Percent motility and age	-0.116	0.20
Percent morphology and age	-0.115	0.20
Oligospermic subjects		
Cytochrome C and age	0.314	0.02
Caspase 3 and age	0.268	0.05
Total antioxidant capacity and age	-0.342	0.01
Sperm concentration and age	-0.180	0.20
Percent motility and age	-0.181	0.20
Percent morphology and age	-0.414	0.002

The R values and levels of statistical significance of pearson correlation of measured variables.

## Discussion

Apoptosis is a major characteristic of sperm cells development, maturation and even to the moment of fertilization of the oocyte. The stimuli that activate apoptosis include toxic metals, environmental toxicants, electromagnetic radiation and chronic medications (10-13). In addition to these factors that stimulate apoptosis in human germ cells, senescence may predispose living organisms to increase apoptosis. In this study markers of apoptosis (caspase 3 and cytochrome C) increased while TAC decreased with increasing age of participants. 

This observation is consistent with previous reports elsewhere (14, 15) even though different markers of apoptosis were evaluated by these authors. Whereas Colin and co-workers (15) measured plasma membrane translocation of phosphatidyserine (marker of early apoptosis), while Uriondo and colleagues (16) evaluated caspase 3, DNA fragmentation and phosphatidyserine in seminal plasma of men of different age groups. They observed that advancing age was significantly and positively correlated with phosphatidyserine and Annexin-v binding. Although correlation for Annexin-V binding was not statistically significant, a clear trend of increased DNA fragmentation was observed in the older study participants with 40 yr as the age threshold (15). 

A significantly higher levels of caspase 3 and DNA fragmentation as well as positive correlation were observed in male subjects evaluated for infertility with >45 yr as age threshold by Ariondo and co-workers (16). These authors suggested that senescence may be associated with significantly higher levels of apoptotic markers in seminal plasma of men with proven fertility and infertility. We previously demonstrated elevated levels of caspase 3 and cytochrome C and lower levels of TAC in infertile men. The higher expression of apoptotic markers was associated with higher levels of oxidative stress indicated by low levels of TAC (17). The relationship between DNA damage and oxidative stress has been reported and the impact of age in this association has also been observed (8, 18). 

Some studies have shown that significantly higher frequencies of sperm cell DNA damage do occur in older men and association between men age and sperm DNA damage in non-clinical samples of active healthy non-smoking subjects and retirees was reported (18). Older men were reported to produce more sperm with DNA damage as a result of age-related increased oxidative stress in their reproductive tracts (19). Although spermatogenesis continues well into old age and some elderly men could father children, fecundity is thought to decline with age (17, 20). The effect of male senescence is rarely considered in the investigation of male factor infertility. It is important to know the effect of male age on sperm DNA damage in men attending infertility clinics because of the risks of abnormal pregnancies, birth defects, infertility and increasing acceptance of modern assisted reproductive technologies (1, 2, 18).

Our results however is not consistent with some authors who stated that apoptotic functions of spermatogenesis may be less effective in older men leading to the release of more sperm with DNA damage (21). This observation was also supported by Brinkworth and Schmid who reported that older mice had lower apoptotic frequencies than young adults (22). Even though apoptosis was identified in the testes of elderly men not many studies have been conducted to compare the rates of apoptosis among men of different ages (18).

The primary trigger for activation of the intrinsic apoptotic cascade is oxidative stress, which could arise from several factors such as poor antioxidant protection within the male genital tract due to dietary deficiencies, age, varicocele and lifestyle factors (22, 23). The tendency to generate elevated levels of reactive oxygen species within the reproductive tract is higher in older men (22). Defective mitochondrial dependent apoptotic cascade is a contributing factor to infertility (24). 

The Sertoli cells aid the regulation of spermatogenesis such that the presence of defective spermatozoa cause Sertoli cell to express FasL which induces cell apoptosis by Fas/FasL pathway. This process helps to maintain equilibrium necessary for normal spermatogenesis (25). However, age exacerbates the generation of ROS and apoptosis and oxidative stress may also compromise fertilizing ability of spermatozoa by reducing the capacity of the spermatozoa to penetrate the vitelline membrane of the oocyte. Some authors have suggested that sperm-oocyte fusion has a biphasic responds depending on the levels of oxidants. At low levels of oxidative stress sperm-oocyte fusion rates are increased but at higher levels of oxidative stress lipid peroxidations are induced in the plasma membrane that may impair sperm-oocyte fusion, probably due to damage to acrosome proteins (26).

We observed decreased levels of sperm indices with increasing age of study participants with a threshold at 40 yr in normospermic but infertile subjects. This observation is consistent with previous studies (2, 12, 27). Although we observed a progressive decrease in percent motility with increasing age, the differences in sperm concentration and percent morphology were insignificant. This is not consistent with other reports (1, 10, 18). Even though sperm quality has declined in the last century (9), paternal age has further made the bad situation worse. Several mechanisms have been used to explain how ageing in men could lead to decline in sperm quality. 

The alterations may be associated with seminal vesicle inadequacy and changes in prostate volume that occur with increasing male age. This observation was further supported by several authors who reported age specific changes in studies that involved large sample size (28, 29). They observed that sperm indices may not change until men attain the age of 35. Experimental studies that investigated the effect of age on the reproductive tract, testes, spermatogenesis and steroidogenesis in ageing laboratory mice observed histological and biochemical alteration associated with age (30). Some of the observed alterations include ultrastructural features such as accumulation of lipofuscin granules, increases in the thickness of the basement membrane and the number of halo cells. Most importantly, alteration in the expression of genes associated with oxidative stress in the epididymis as a result of age was reported (30). 

Apoptosis has a significant correlation with infertility in men. Increased apoptosis adversely affect sperm parameters; progressive motility and concentration. Elevated apoptosis makes spermatozoa functionally incompetent with disorder in the tail of spermatozoa, a, process that was attributed to positive annex that makes the movement of the tail difficult (31). The mechanisms of apoptosis occur via cell receptors and if the process is prolonged, the cells die without inflammation and the components of the dying cells are engulfed and recycled by macrophages (32).

## Conclusion

In conclusion, the measured apoptotic markers increased with increasing age while TAC and sperm indices decreased with increasing age of subjects evaluated for infertility. The increase in apoptosis due to age may have adverse implications for reproductive health. Although the measured apoptosis and oxidative stress markers correlated with age in normozospermia, the effect on sperm indices was severe among oligospermia compare to normozospermia. Therefore, these markers may be assayed in aged men attending fertility clinics, and they should be counseled and educated about the risks involved in making babies at old age.
